# Cord serum metabolic signatures of future progression to immune-mediated diseases

**DOI:** 10.1016/j.isci.2023.106268

**Published:** 2023-02-25

**Authors:** Tuulia Hyötyläinen, Bagavathy Shanmugam Karthikeyan, Tannaz Ghaffarzadegan, Eric W. Triplett, Matej Orešič, Johnny Ludvigsson

**Affiliations:** 1 School of Science and Technology, Örebro University, 702 81 Örebro, Sweden; 2 School of Medical Sciences, Örebro University, 702 81 Örebro, Sweden; 3 Department of Microbiology and Cell Science, Institute of Food and Agricultural Sciences University of Florida, Gainesville, FL 32611-0700, USA; 4 Turku Bioscience Centre, University of Turku and Åbo Akademi University, 20520 Turku, Finland; 5 Crown Princess Victoria’s Children’s Hospital and Division of Pediatrics, Department of Biomedical and Clinical Sciences, Linköping University, 581 85 Linköping, Sweden

**Keywords:** Health sciences, Human metabolism, Immunology, Lipidomics, Metabolomics

## Abstract

Previous prospective studies suggest that progression to autoimmune diseases is preceded by metabolic dysregulation, but it is not clear which metabolic changes are disease-specific and which are common across multiple immune-mediated diseases. Here we investigated metabolic profiles in cord serum in a general population cohort (All Babies In Southeast Sweden; ABIS), comprising infants who progressed to one or more immune-mediated diseases later in life: type 1 diabetes (n = 12), celiac disease (n = 28), juvenile idiopathic arthritis (n = 9), inflammatory bowel disease (n = 7), and hypothyroidism (n = 6); and matched controls (n = 270). We observed elevated levels of multiple triacylglycerols (TGs) an alteration in several gut microbiota related metabolites in the autoimmune groups. The most distinct differences were observed in those infants who later developed HT. The specific similarities observed in metabolic profiles across autoimmune diseases suggest that they share specific common metabolic phenotypes at birth that contrast with those of healthy controls.

## Highlights

• Cord serum metabolomics performed in a general population prospective birth cohort
• Primary outcomes of interest were later progression to specific autoimmune diseases
• Many similarities in metabolic profiles were detected across the autoimmune diseases
• Later hypothyroidism had the most distinct metabolic profile, with increased lipids

## Graphical abstract

**Figure:**
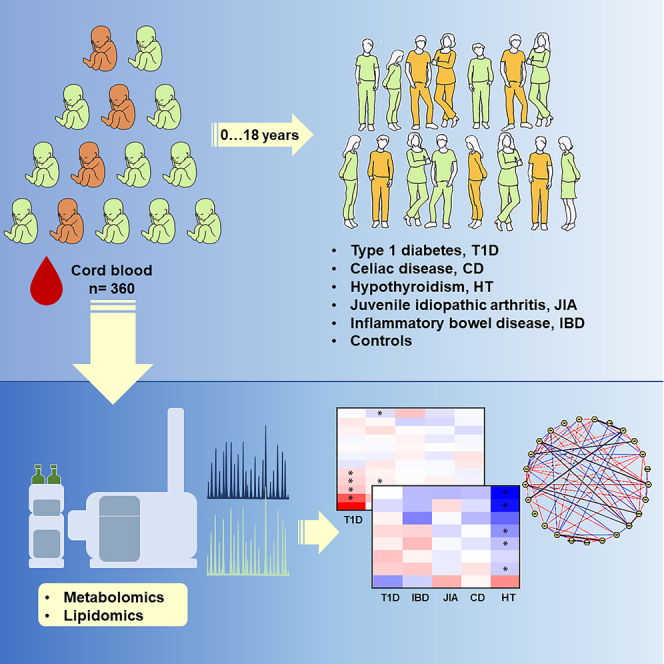

## Introduction

Autoimmunity is a complex process contributing to widespread functional decline that affects multiple organs and tissues. Overall, over 80 autoimmune diseases have been identified including, among the most common ones, type 1 diabetes (T1D), multiple sclerosis, celiac disease (CD), inflammatory bowel disease (IBD), and rheumatoid arthritis (RA).^1^ Several of the autoimmune diseases are manifested in childhood. The prevalence and incidence of several of these autoimmune diseases have increased over the last decades.^2^^,^^3^^,^^4^^,^^5^ The pathogenesis of most of the autoimmune diseases is, however, generally not fully characterized. It has been suggested that both genetic predisposition and environmental factors, and their mutual interactions, play a significant role in the disease pathogenesis.^6^^,^^7^

Many autoimmune diseases share common risk factors or pathogenic mechanisms. For example, T1D and CD share common predisposing alleles in the class II HLA-region.^8^^,^^9^ Approximately 6% of patients with T1D also develop clinical CD^10^ whereas subjects with CD are at risk for developing T1D before age 20,^10^ T1D, multiple sclerosis (MScl), and RA are also classified as T cell-mediated autoimmune diseases.^11^ Importantly, it has been shown that fundamental processes underlying T cell functionality are linked to changes in the cellular metabolic programs.^12^ External perturbation of key metabolic processes may impair T cell activation, differentiation, and cytokine production. We have also shown that differentiating human CD4^+^T-cells have subset-specific differences in glycosphingolipid pathways.^13^

Abnormal metabolism is a common feature of several autoimmune diseases, which occurs before the onset of clinical disease, including in T1D,^14^^,^^15^ CD,^16^^,^^17^^,^^18^ and IBD.^9^ Changes in specific phospholipids and amino acids have been reported at birth in genetically disposed children who progressed to islet autoimmunity and T1D later in life.^14^ In adolescents and adults, similarly as in children, metabolic dysregulation related to altered phospholipid profiles and alteration in steroidogenesis, bile acid biosynthesis and sugar metabolism have been reported.^19^ In future CD, altered levels of phospholipids and triacylglycerols have been detected already before the infants had been exposed to gluten.^9^ In pediatric IBD, alteration in metabolome, including phospholipids, has been reported,^20^ with similar changes being reported also in adults including downregulation of alky lether phospholipids such as plasmalogens.^21^ In other autoimmune diseases, dysregulated amino acid, central carbon, and phospholipid metabolism have been associated with rheumatoid arthritis.^22^^,^^23^ In autoimmune thyroid disease, altered amino acid pathways, primary bile acid biosynthesis, and steroid hormone biosynthesis have been identified.^24^^,^^25^ In adult CD, recent meta-analysis reported conflicting results, however, most studies were focused on a limited set of metabolites, such as short-chain fatty acids and ketogenic metabolites^26^ and the adult CD is highly heterogeneous. Overall, especially in children, current data thus suggest that there may be some commonalities between metabolic signatures preceding different autoimmune diseases. However, at present there are very few studies comparing common and specific metabolic patters preceding multiple autoimmune diseases.

Herein, we investigate cord serum metabolomes in a general population cohort (All Babies In Southeast Sweden; ABIS),^27^ comprising children who later progressed to one or more immune-mediated diseases (T1D, CD, juvenile RA [JIA], IBD, hypothyroidism [HT]), and matched controls. We studied the metabolic changes across all autoimmune mediated disease groups, looking at the overall metabolic changes in those subjects later developing a specific disease. We also investigated whether maternal lifestyle factors had an impact on the observed changes, and further investigated the association of the specific HLA-conferred risk factors with metabolic profiles.

## Results

### Metabolic profiles in cord blood

A total of 545 lipids and 3,417 polar/semipolar metabolites were detected in cord serum, of which 201 lipids and 120 metabolites were identified at the level 1 and 2 and quantified, and additional 20 metabolites were identified at the level 3 (Metabolomics Standard Initiative^28^ as marked in Tables S1 and S2). To investigate global changes of metabolomes across the study groups (Table 1), including also the unidentified compounds, we first performed model-based clustering for the two datasets separately, with the clustering resulting in 8 lipid clusters (LC) and 12 polar metabolite clusters (PC) (Table 2).

**Table 1:** Demographic characteristics of the study cohort. Values shown as means (standard deviation), unless noted otherwise

	CD	IBD	JIA	HT	T1D	Controls
N (F/M)	28 (9/19)	7 (5/2)	9 (3/6)	6 (0/6)	12 (8/4)	270 (124/152)
Gestational age (weeks)	40 (1.7)	40 (1.7)	40 (1.1)	39 (1.8)	39 (1.0)	40 (1.5)
Birth weight (g)	3705 (586)^a^	3540 (753)	3640 (487)	3163 (488)^a^	3745 (512)	3580 (503)
Maternal age (year)	30.0 (4.6)	31.0 (3.0)	27.0 (6.1)	30.5 (6.0)	30.0 (6.5)	29.0 (4.7)
Maternal BMI (kg/m^2^)	23.0 (4.7)	22.3 (2.4)	23.7 (1.5)	23.4 (3.5)	22.9 (4.8)	22.9 (3.8)
Delivery (vaginal/cesarean/b)	22/3/3	4/0/3	7/2/0	5/0/1	7/3/2	231/23/48
Age of diagnosis (years)	11.5 (5.7)	16 (1.6)	15 (5.3)	16 (1.6)	13.5 (3.3)	NA

a Significant difference in comparison with the control group.

b not available for all subjects

**Table 2:** Description of lipid (LC) and polar metabolite (PC) clusters

Cluster	Main classes of compounds	Specific examples
LC1	LPC, SM, Cer	SM(42:3), LPC(22:5), Cer(d18:½4:0)
LC2	PC, PC_O	PC(40:8), PC(40:6), PC(O-40:4)
LC3	CE, Lac/HexCer, PC, PI, SM	CE(18:0), CE(18:1), Hexcer(d18:½4:0)
LC4	PC_PUFA, LPC_PUFA	LPC(18:2), LPC(20:4), PC(38:4)
LC5	TG_SFA	TG(14:0/16:0/18:1),TG(16:0/16:0/16:0), TG(50:0)
LC6	TG_MUFA, TG_PUFA	TG(58:9), TG(18:1/18:½2:6), TG(58:6)
LC7	Unknowns	Putative identifications: TGs
LC8	Unknowns	Putative identifications: various phospholipids
PC1	Bile acids, microbial metabolites	CA, CDCA, GCA, 3-indoleacetic acid
PC2	Amino acids	Valine, Phenylalanine, lysine, serine
PC3	Free fatty acids, lipids	Arachidonic acid, 16-Hydroxypalmitate, LPC(17:0)
PC4	Unknowns, highly polar compounds	
PC5	Free fatty acids, lipids	C16:1, C18:2, linoleic acid
PC6	Unknowns	
PC7	Unknowns	
PC8	Unknowns	
PC9	Unknowns	
PC10	Unknowns	
PC11	Unknowns, highly polar compounds	Putative identifications: exogeneous compounds
PC12	Unknowns	

**Figure 1:**
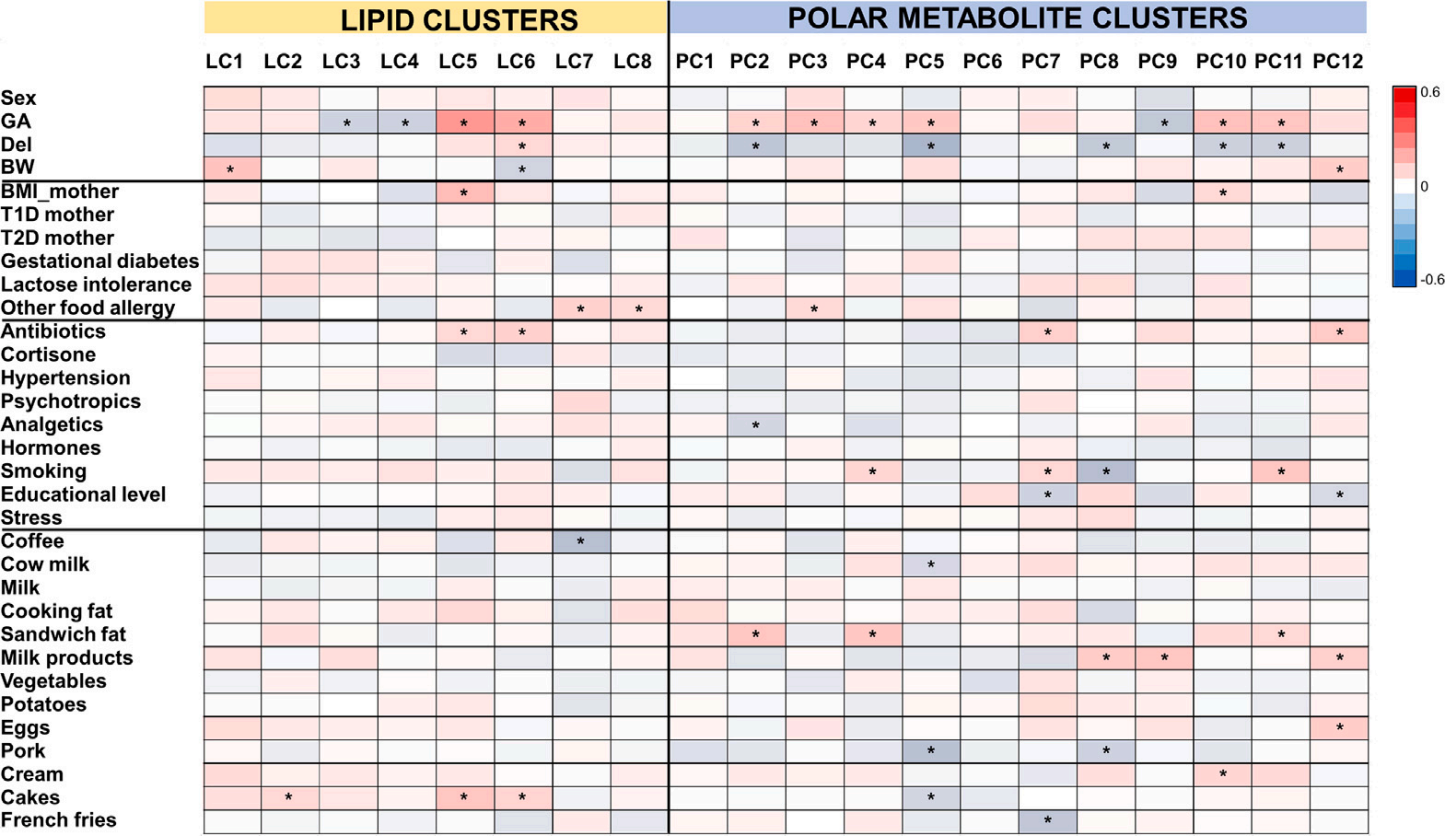
Associations of various demographic and lifestyle factors, and food intake with metabolome Spearman correlations shown between lipid and polar metabolite clusters and the metadata. ∗p<0.05. Abbreviations: BW, birth weight; Del, delivery mode (cesarean versus vaginal); GA, gestational age; Sex (female versus male); T1D, type 1 diabetes; T2D, type 2 diabetes.

We first investigated whether the gestational age, sex, birth weight or maternal factors (including BMI, maternal age, maternal diagnosis, dietary patterns) had an impact on the metabolome. Out of these parameters, gestational age and birth weight showed the most significant association with metabolite clusters (Figure 1) and several individual metabolites (Table S1). Also, maternal age showed associations with the lipid and metabolite clusters. Maternal BMI and diet had modest impact on cord blood metabolome, the former via positive associations with TGs containing saturated fatty acyls. The latter had weak impact on the cord blood metabolome (R below ±0.25), except for three known metabolites of coffee that showed significant association between maternal coffee consumption and cord blood levels of these metabolites (R= 0.38–0.81, p<0.0001). Among maternal diagnoses, other food allergies than lactose intolerance or nut allergy showed significant associations with clusters LC7, LC8 and PC3, smoking with four polar metabolite clusters (PC4, PC7, PC8 and PC11), use of antibiotics with LC5, LC6, PC7 and PC12 and educational level with PC7 and PC12. The latter may be attributed to the negative association between the educational level and smoking, and associations between educational level and diet (negative association between educational level and vegetables in the diet, positive association with eating French fries).

For further data analyses, we investigated the impact of adjustment with maternal age, maternal BMI, gestational age, and birth weight. Among these factors, maternal age, gestational age and birth weight had an impact on the results, and for further data analysis, the data were adjusted with these three factors.

### Autoimmune diseases share similar metabolic profiles already at birth

**Figure 2:**
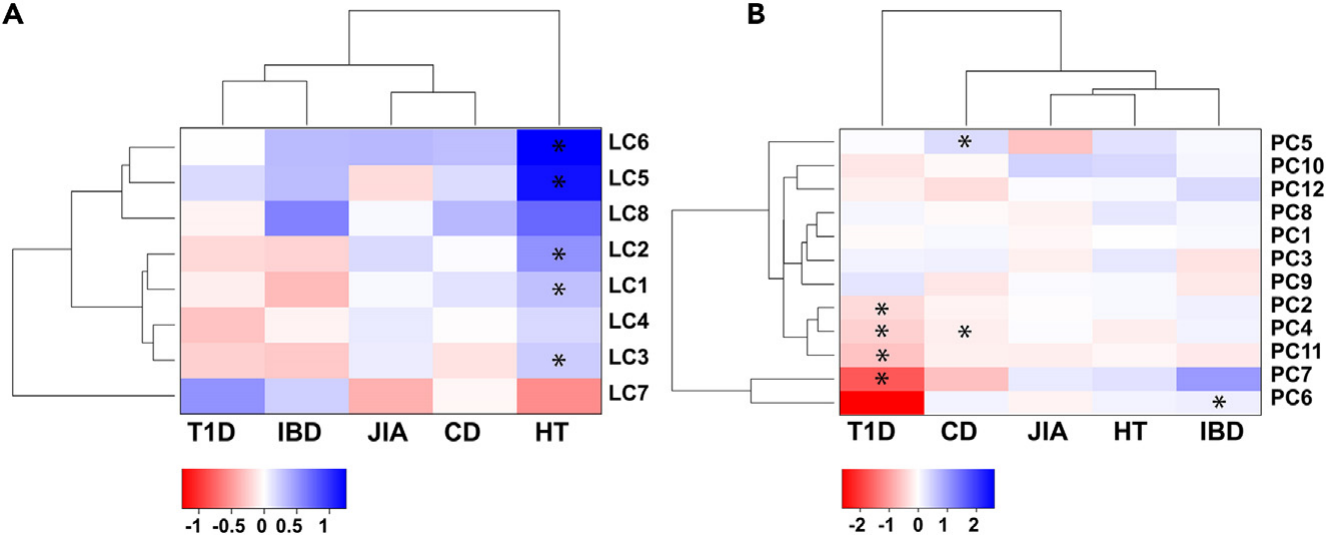
Comparison of different autoimmune disease groups and controls at the metabolite cluster level (A) Lipid clusters and (B) metabolite clusters. Logfold change (FC) with ∗p.adjusted<0.05. Cluster descriptions are provided in Table 2.

We observed significant differences between the control group and the different diagnostic groups, both at the level of lipid and metabolite clusters as well as at the level of individual metabolites (Figure 2, Tables S2 and S3), after adjustment for gestational age, birth weight and maternal age. We investigated the differences both at the level of individual disease diagnosis as well as by pooling all autoimmune cases together, excluding the HT group as it appeared to be an outlier among the disease groups.

Among the individual diagnostic groups, the subjects who later developed HT differed most significantly from the control group. Five of the eight lipid clusters showed significantly upregulated levels in HT compared to controls. Overall, all disease groups showed a trend of upregulation of lipid clusters LC5, LC6 and LC8, although the difference between the groups compared with controls was only significant for HT. These three lipid clusters are composed of mainly triacyclglycerols (TGs). Overall, T1D and IBD clustered together with similar trend over multiple lipid clusters. Similarly, CD and JIA clustered together. On metabolic cluster level, T1D showed significant differences in comparison with control group in PC2, PC4, PC7 and PC11. The CD group showed significant differences in PC4 and PC5, whereas the IBD group showed significant differences in PC6. PC2 includes mainly amino acids, PC5 includes mainly on free fatty acids, and other polar lipids, PC4 and PC11 consist of mainly unidentified metabolites, which based on their chromatographic behavior are highly polar small metabolites, whereas PC7 includes semipolar compounds putatively identified as free fatty acids and polar lipid derivatives.

Among the individual metabolites, 17 lipids and seven polar metabolites were different between the control and case groups at the level of nominal p values; however, none reached statistical significance after FDR correction. These lipids were mainly TGs comprising saturated fatty acyls, whereas the polar metabolites included mainly secondary bile acids, one short-chain fatty acid, and two amino acids. In specific diseases, we observed changes particularly in HT in lipids, with upregulated levels of large number of lipids (TGs, SMs, and several other phospholipids) and downregulation of dehydroepiandrosterone sulfate. In CD, we observed a trend of decreased levels of phospholipids (PC, SM), secondary bile acid UDCA and serine and increased TGs, isovaleric acid and C20:5. In IBD, trend of decreased levels of ether PCs and some other phospholipids were observed as well as increased levels of isovaleric and isocapric acid. In JIA, the main difference was in TGs, with increased levels compared to controls, and also differences in several gut microbiota-related metabolites. In T1D, we observed decreased levels of phospholipids, including PCs and SMs, and downregulation of CDCA and fructose.

### The autoimmune cases showed difference in metabolic co-regulation

**Figure 3:**
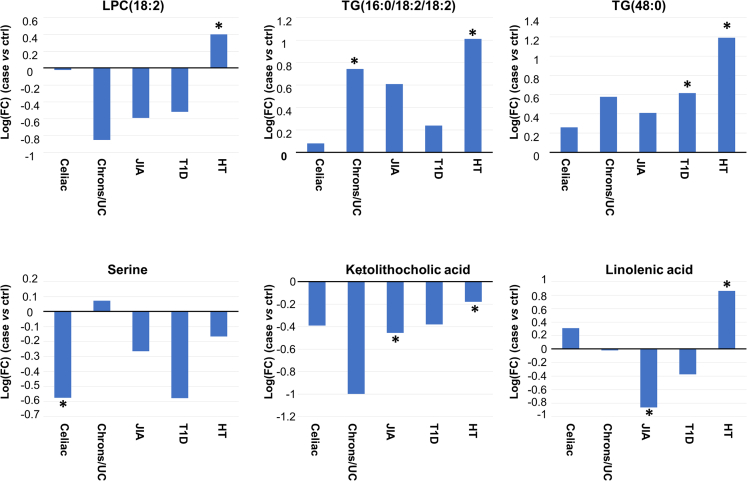
Relative levels of selected metabolites across the study groups ∗p.adjusted<0.05. Linear models adjusted for for maternal age, birth weight and gestational age.

Next, we investigated the interplay of the lipid and metabolite clusters and clinical features in autoimmune cases and control groups separately (Figure 3A) as well as those lipids and polar metabolites that showed significant differences (Figure 3B). In autoimmune group, the gestational age showed negative association with PC9 whereas this association was much weaker in control group. The birth weight showed negative association with LC6 in the autoimmune group whereas this association was absent in the control group. We also observed clear differences between the case and control groups in metabolite and lipid cluster mutual associations.

### Pathway analysis reveals alteration in lipid metabolism

**Figure 4:**
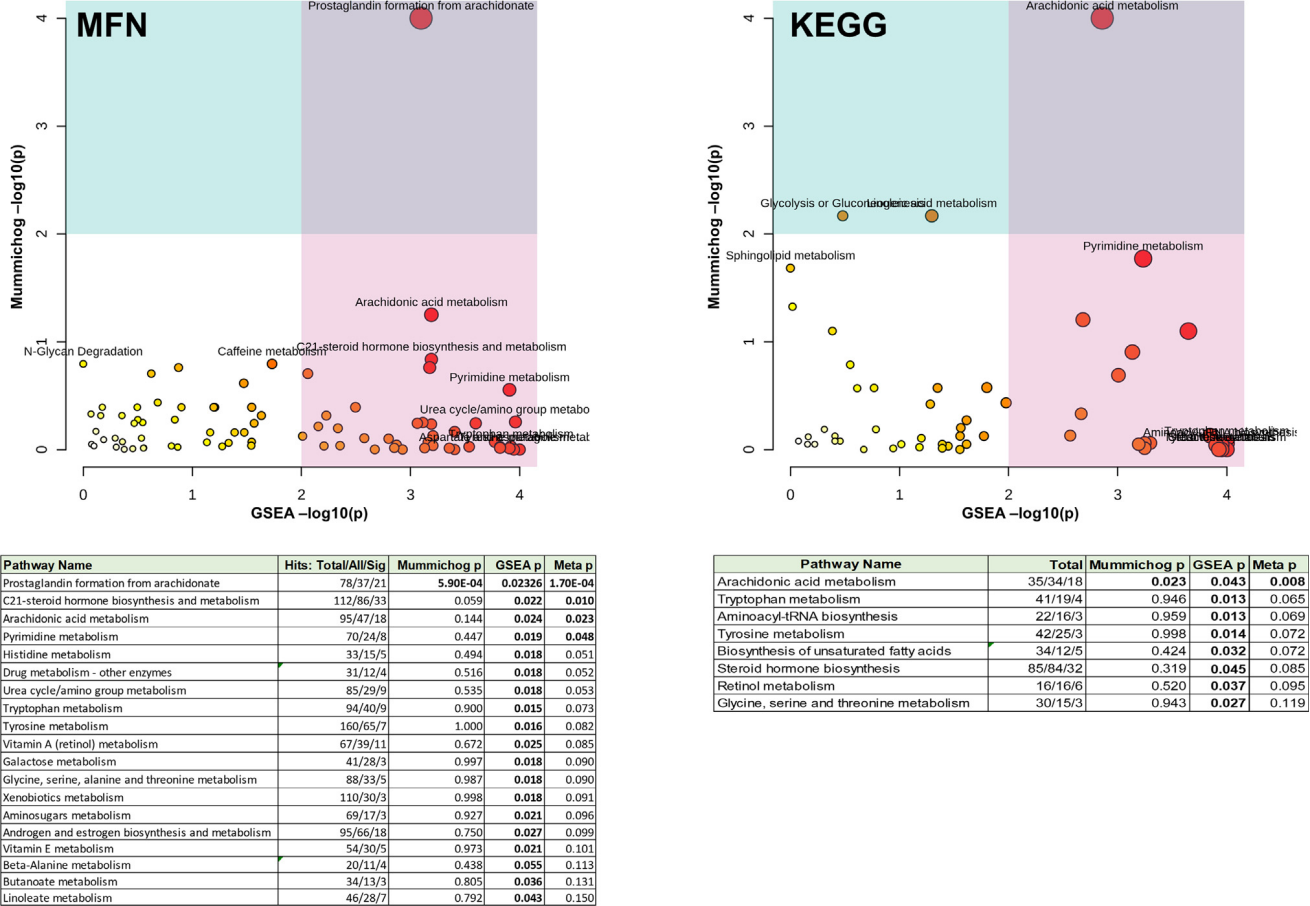
Pathway analysis comparing cases (without HT group) versus controls The upper panels show pvalues using the MFN (left) and KEGG (right) pathway maps, using Mummichog (yaxis) and GSEA (xaxis) pathway analysis methods. Size of the circle corresponds to the pathway impact value. The corresponding tables with number of metabolites in the pathways (total number/hits/significant hits) and pvalues shown under these panels. Abbreviations: Meta pvalue calculated by combined GSEA and Mummichog score; Sig, significant.

Pathway analyses were performed by comparing controls against autoimmune mediated diseases grouped together (CD, T1D, JIA, IBD) using both Mummichog and GeneSet Enrichment Analysis (GSEA) algorithms for the pathway analyses to increase their robustness. We further filtered the results based on the number of metabolites detected in each pathway and the number of significant hits. The results indicated that the autoimmunity was associated with multiple pathways including arachidonic acid metabolism, steroid and tryptophan metabolism (Figure 4).

**Figure 5:**
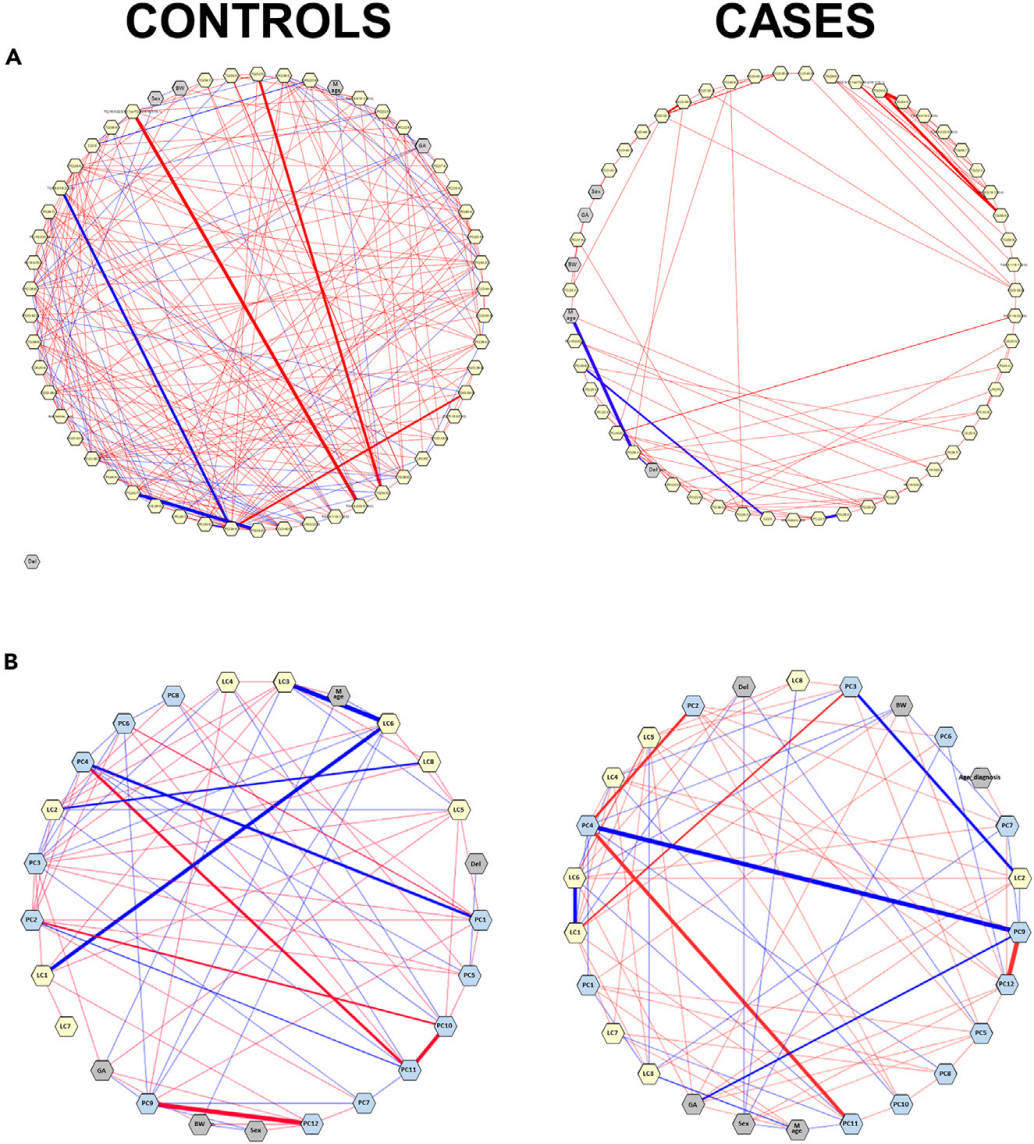
Partial correlation network analysis, done separately for controls and cases (excluding HT) Here, each node represents a metabolite, metabolite cluster, or a clinical parameter (gray color). Each edge represents the strength of partial correlation between two compounds/parameters after conditioning on all other compounds in the datasets. Edge weights represent the partial correlation coefficients, with Edge colors: blue color for negative correlations and red for positive correlations, the thickness of the line shows the strength of the correlation. Edge ranges adjusted between ±0.22 to 1. (A) Arachidonic acid and DHA containing lipids in yellow color, with partial correlations p<0.1. (B) Network on the level of lipid (yellow color) and polar metabolite clusters (blue color).

Next, we selected those lipids that contain either arachidonic acid (AA) or docosahexaenoic acid (DHA), as these lipids have shown to be a crucial role in the development of the infant immune system.^29^ We then examined the difference between the controls and autoimmune groups, by applying a partial correlation analysis (Figures 5A and 5B). The intra-lipid correlations were clearly weaker in the autoimmune group when compared with the control group (Figure 5A), although there was no significant difference in the partial correlation between lipid classes on the two groups (Figure 5B).

### HLA risk is associated with changes in amino acid and PUFA

**Figure 6:**
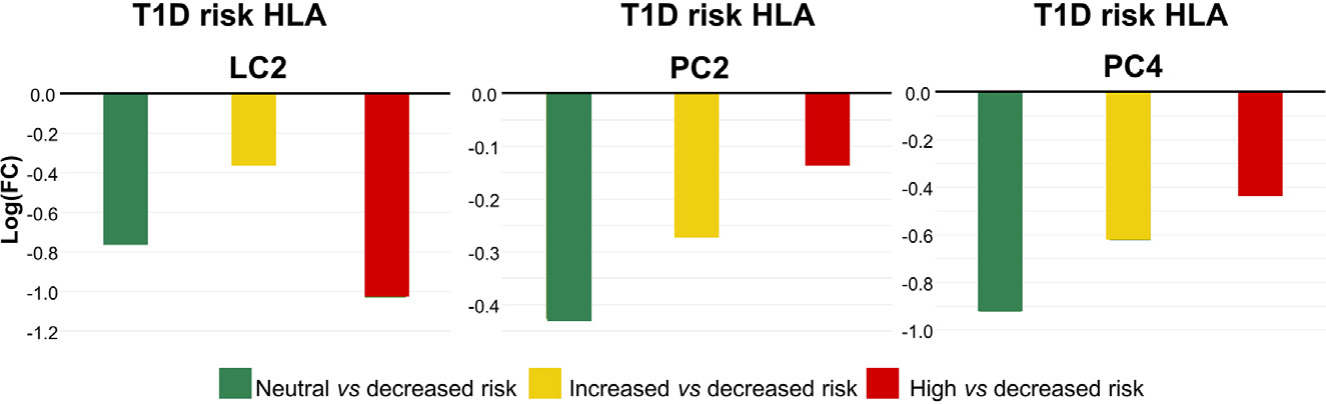
Impact of HLA-conferred risk for T1D on metabolic profiles LIMMA model, adjusted with maternal age, birth weight and gestational age, logarithmic fold changes between cases with neutral versus decreased risk (green), increased versus decreased risk (yellow) and high versus decreased risk (red) for lipid cluster 2 (p = 0.018) and polar metabolite clusters 2 (p= 0.041) and 4 (p = 0.015).

Next, we investigated the association between HLA risk genotype and metabolite profiles, both at the cluster and individual metabolite level by using a linear regression model. For T1D, the risk genotypes were classified as decreased, neutral, increased, and high risk while in CD, the groups were very low, low, and moderate. The T1D risk type was associated with LC2, PC2 and PC4, the latter two showing reduced levels in comparison with the decreased genotype versus neutral, increased, and high-risk genotypes (Figure 6). At the level of individual lipids and polar metabolites, large number of phospholipids, both PCs and SMs, particularly those PCs with PUFA showed similar trends, as well as AA and DHA, i.e., with reduced levels with increasing risk HLA risk genotype (Table S4). For CD, the metabolic profiles did not show associations with the risk genotype.

## Discussion

We performed untargeted metabolomics analyses to obtain a comprehensive picture of metabolic profiles in cord blood samples in infants who later developed autoimmune diseases. The similarities in metabolic profiles, particularly across T1D, JIA, IBD, CD, suggests that the diseases share common metabolic alteration already at birth, i.e., years before the onset of the disease. As a common feature, we observed elevated levels of multiple classes of TGs, including both saturated and polyunsaturated fatty acid containing TGs. In addition, multiple gut microbiota related metabolites, such as secondary bile acids UDCA and ketolithocholic acid, were altered in the autoimmune groups. The most significant pathways impacted were related to arachidonic acid derived fatty acid metabolism (prostaglandin and leukotriene metabolism) and steroid hormone metabolism. Of individual diseases, most distinct differences were observed in those infants who later developed HT, showing significantly increased levels of large number of lipids.

We also observed that phospholipids, particularly PUFA containing lipids, as well as free fatty acids AA and DHA were associated with HLA-conferred disease risk, with decreased levels of this type of lipids with increasing genotype risk profile. The AA pathway has been shown to play a key role in inflammatory processes.^30^^,^^31^ Indeed, chronic inflammation is known to be an underlying cause of multiple diseases, such as metabolic syndrome, type 2 diabetes, non-alcoholic fatty liver disease, hypertension, cardiovascular disease, and autoimmune diseases.^32^ The role of arachidonic acid in inflammation is related to the production of oxylipins, which are oxygenated lipid mediators that promote or resolve inflammation.^30^ The AA-related oxylipins are usually considered to be inflammatory, proliferative and vasoconstrictive.^30^ Elevated plasma arachidonic acid to docosahexaenoic acid ratios have also been associated with increased risk of IA in the Finnish Type 1 Diabetes Prediction and Prevention Study (DIPP) birth cohort.^33^^,^^34^ The AA-related oxylipins have also been shown to be associated with increased risk of type 1 diabetes risk in Diabetes Autoimmunity Study in the Young (DAISY) cohort.^31^ Also in adult subjects with IBD, PUFA dysregulation has been suggested to be associated in the bowel inflammation process through eicosanoids, derived from AA corresponding to increased colonic inflammatory cytokines and increased serum fatty acids.^35^ Similarly, in rheumatoid arthritis, AA metabolism has been suggested to play an important role in the disease manifestation.^22^

Currently, there are no previous studies that compared the metabolic patterns in cord-blood of children who later developed different autoimmune diseases in a general population-based set-up, or studies that would have linked the HLA risk type with metabolic profiles in infants. There are multiple studies, including our earlier studies on predictive metabolic patterns of T1D^14^^,^^36^ and CD,^9^ however, these have been done in a genetically high-risk cohorts. We did observe some similarities with the current study and our earlier results, particularly related to changes in CD. However, it should be noted that the current cohort has distinct differences related to previous studies, particularly as in the current cohort the median age of diagnosis was 15 years, whereas in the high risk T1D and CD cohorts we have investigated earlier the median age of diagnosis was much lower (<10 years). Our results were also in agreement of published results on metabolomic changes reported in patients with rheumatoid arthritis which have reported that children with active JIA had higher plasma triglyceride concentrations compared to healthy control subjects.^37^^,^^38^ Adult subjects with rheumatoid arthritis, on the other hand, have shown to have lower levels of multiple LPCs, which were further correlated with interleukin-6 and disease activity indices.^23^

Overall, our study suggests that there are shared metabolic characteristics across multiple autoimmune diseases, plausibly because of shared physiopathologic mechanisms, genetic and environmental factors because of autoimmune tautology. However, more mechanistic studies are required to elucidate the pathways responsible for the disease development, and the factors contributing to the process.

This study in a general-population prospective birth cohort indicates that future autoimmune diseases share several common features in metabolic profiles at birth. The causes of these common features and their relevance for disease pathogenesis are yet to be elucidated. Given these metabolic profiles are detected already at birth, likely causes are attributed to maternal diet and other environmental exposures.

### Limitations of the study

We acknowledge limitations of the study. The number of subjects within each disease group was low. This is an inherent limitation of general population study setting when studying the diseases with low incidence. As a strength of such setting, the study is not limited to populations with HLA-conferred risk of specific diseases, thus allowing for comparative studies across the different diseases. Although the analytical coverage of the metabolites was comprehensive, we could not identify all metabolites detected. However, the pathway analysis tool does include the whole data and it also includes pathway to identify the unknown compounds, thus giving a representative view of the metabolic changes at the pathway level.

## STAR★Methods

### Key resources table

**Table:** 

REAGENT or RESOURCE	SOURCE	IDENTIFIER
**Chemicals, Peptides and Recombinant Proteins**		

2-diheptadecanoyl-sn-glycero-3- phosphoethanolamine (PE(17:0/17:0))	Avanti Polar Lipids	Cat#830756
N-heptadecanoyl-D-erythro- sphingosylphosphorylcholine (SM(d18:1/17:0))	Avanti Polar Lipids	Cat#860585
1-stearoyl-2-hydroxy-sn-glycero-3- phosphocholine (LPC(18:0))	Avanti Polar Lipids	Cat#855775
2-diheptadecanoyl-sn-glycero-3- phosphocholine (PC(17:0/17:0))	Avanti Polar Lipids	Cat#850360
1-heptadecanoyl-2-hydroxy-sn-glycero-3- phosphocholine (LPC(17:0))	Avanti Polar Lipids	Cat#855676
2-Dioctadecanoyl--sn-glycero-3- phosphocholine (PC(18:0/18:0))	Avanti Polar Lipids	Cat#850333
1-Hexadecanoyl-2-oleoyl-sn-glycero-3- phosphocholine (PC(16:0/18:1)	Avanti Polar Lipids	Cat#850457
1-(9Z-octadecenoyl)-sn-glycero-3- phosphoethanolamine (LPE(18:1))	Avanti Polar Lipids	Cat#850456
1-Palmitoyl-2-Hydroxy-sn-Glycero-3- Phosphatidylcholine (LPC(16:0))	Avanti Polar Lipids	Cat#846725
triheptadecanoylglycerol (TG(17:0/17:0/17:0))	Larodan	Cat#33-1700
trihexadecanoalglycerol (TG(16:0/16:0/16:0))	Larodan	Cat#33-1610
1-stearoyl-2-linoleoyl-sn-glycerol (DG(18:0/18:2))	Avanti Polar Lipids	Cat#855675
3-trioctadecanoylglycerol (TG(18:0/18:0/18:0))	Larodan	Cat#33-1810
3β-Hydroxy-5-cholestene-3-linoleate (ChoE(18:2))	Larodan	Cat#64-1802
1-hexadecyl-2-(9Z-octadecenoyl)-sn-glycero-3-phosphocholine (PC(16:0e/18:1(9Z)))	Avanti Polar Lipids	Cat#800817
1-(1Z-octadecanyl)-2-(9Z-octadecenoyl)- sn-glycero-3-phosphocholine (PC(18:0p/18:1(9Z)))	Avanti Polar Lipids	Cat#878112
1-oleoyl-2-hydroxy-sn-glycero-3- phosphocholine (LPC(18:1))	Larodan	Cat#38-1801
1-palmitoyl-2-oleoyl-sn-glycero-3- phosphoethanolamine (PE(16:0/18:1))	Avanti Polar Lipids	Cat#852467
3β-hydroxy-5-cholestene-3-stearate (ChoE(18:0))	Larodan	Cat#64-1800
1-palmitoyl-d31-2-oleoyl-sn-glycero-3- phosphocholine (PC(16:0/d31/18:1))	Avanti Polar Lipids	Cat#850757
2-diheptadecanoyl-sn-glycero-3- phosphoethanolamine (PE(17:0/17:0))	Avanti Polar Lipids	Cat#830756
N-heptadecanoyl-D-erythro- sphingosylphosphorylcholine (SM(d18:1/17:0))	Avanti Polar Lipids	Cat#860585
1-stearoyl-2-hydroxy-sn-glycero-3- phosphocholine (LPC(18:0))	Avanti Polar Lipids	Cat#855775
beta-Muricholic acid	Steraloids	Cat# C1895-000
Chenodeoxycholic acid	Sigma-Aldrich	Cat# C1050000
Cholic acid	Sigma-Aldrich	Cat# C2158000
Deoxycholic acid	Sigma-Aldrich	Cat# 700197P
Glycochenodeoxycholic acid	Sigma-Aldrich	Cat# 700266P
Glycocholic acid	Sigma-Aldrich	Cat# 700265P
Glycodehydrocholic acid	Steraloids	Cat# C2020-000
Glycodeoxycholic acid	Glycocholic acid	Sigma-Aldrich
Glycohyocholic acid	Steraloids	Cat#C1860-000
Glycohyodeoxycholic acid	Steraloids	Cat# C0867-000
Glycolitocholic acid	Sigma-Aldrich	Cat# 700268P
Glycoursodeoxycholic acid	Sigma-Aldrich	Cat# 06863
Hyocholic acid	Steraloids	Cat# C1850-000
Hyodeoxycholic acid	Steraloids	Cat# C0860-000
Litocholic acid	Sigma-Aldrich	Cat#700218P
alpha-Muricholic acid	Steraloids	Cat# C1891-000
Tauro-alpha-muricholic acid	Steraloids	Cat# C1893-000
Tauro-beta-muricholic acid	Steraloids	Cat# C1899-000
Taurochenodeoxycholic acid	Sigma-Aldrich	Cat# 700249P
Taurocholic acid	Sigma-Aldrich	Cat# T9034
Taurodehydrocholic acid	Sigma-Aldrich	Cat# 700242P
Taurodeoxycholic acid	Sigma-Aldrich	Cat# 700250P
Taurohyodeoxycholic acid	Sigma-Aldrich	Cat# 700248P
Taurolitocholic acid	Sigma-Aldrich	Cat# 700252P
Tauro-omega-muricholic acid	Steraloids	Cat# C1889-000
Tauroursodeoxycholic acid	Sigma-Aldrich	Cat# 580549
Trihydroxycholestanoic acid	Avanti Polar Lipids	Cat# 700070P
Fumaric acid	Sigma-Aldrich	Cat#47910
Glutamic acid	Sigma-Aldrich	Cat# G0355000
Aspartic acid	Sigma-Aldrich	Cat# A1330000
Serine	Sigma-Aldrich	Cat# S4500
Threonine	Sigma-Aldrich	Cat# PHR1242
Glutamine	Sigma-Aldrich	Cat# G3126
Proline	Sigma-Aldrich	Cat#V0500
Valine	Sigma-Aldrich	Cat# PHR1172
Lysine	Sigma-Aldrich	Cat# L5501
Methionine	Sigma-Aldrich	Cat# M0960000
Syringic acid	Sigma-Aldrich	Cat# 63627
Isoleucine	Sigma-Aldrich	Cat# I2752
Leucine	Sigma-Aldrich	Cat# L8000
Malic Acid	Sigma-Aldrich	Cat# PHR1273
Phenylalanine	Sigma-Aldrich	Cat# P2126
Ferulic acid	Sigma-Aldrich	Cat# Y0001013
Citric acid	Sigma-Aldrich	Cat# C7129
Tryptophan	Sigma-Aldrich	Cat# 93659
3-Indoleacetic acid	Sigma-Aldrich	Cat#I3750
3-Hydroxybutyric acid	Sigma-Aldrich	Cat#52017
Isovaleric acid	Sigma-Aldrich	Cat# 78651
Indole-3-propionic acid	Sigma-Aldrich	Cat# 57400
Salicylic acid	Sigma-Aldrich	Cat# 247588
Isocaproic acid	Sigma-Aldrich	Cat# 277827
Decanoic acid	Sigma-Aldrich	Cat# C1875
Myristic acid	Sigma-Aldrich	Cat# 70079
Linolenic acid	Sigma-Aldrich	Cat# 62160
Palmitoleic acid	Sigma-Aldrich	Cat# 76169
Linoleic acid	Sigma-Aldrich	Cat# 62230
Eicosapentaenoic acid	Sigma-Aldrich	Cat# 44864
Palmitic acid	Sigma-Aldrich	Cat# P0500
Oleic acid	Sigma-Aldrich	Cat# 75090
Stearic acid	Sigma-Aldrich	Cat# S4751
Arachidic acid	Sigma-Aldrich	Cat# 39383
[D4]- Glycoursodeoxycholic acid	Bionordica	Cat#31309
[D4]- Glycocholic acid	Bionordica	Cat#21889
[D4]- Ursodeoxycholic acid	Bionordica	Cat#21892
[D4]- Glycochenodeoxycholic acid	Bionordica	Cat#21890
[D4]- Cholic acid	Bionordica	Cat#20849
[D4]- Glycolitocholic acid	Bionordica	Cat#31308
[D4]- Chenodeoxycholic acid	Bionordica	Cat#20848
[D4]- Deoxycholic acid	Bionordica	Cat#20851
[D4]- Litocholic acid		Cat#20831
Valine-d8	Sigma-Aldrich	Cat#486027
Glutamic acid-d5	Sigma-Aldrich	Cat# 631973
Succinic acid-d4	Sigma-Aldrich	Cat# 293075
Heptadecanoic acid	Sigma-Aldrich	Cat# H3500
Lactic acid-d3	Sigma-Aldrich	Cat# 616567
Citric acid-d4	Sigma-Aldrich	Cat# 485438
Arginine-d7	Sigma-Aldrich	Cat# 776408
Tryptophan-d5	Sigma-Aldrich	Cat# 615862
Glutamine-d5	Sigma-Aldrich	Cat# 616303

### Resource availability

#### Lead contact

Further information and requests for resources and reagents should be directed to and will be fulfilled by the lead contact: Tuulia Hyötyläinen (tuulia.hyotylainen@oru.se).

#### Materials availability

This study did not generate new unique reagents.

### Method details

Cord serum samples from a All Babies In Southeast Swedecohort (ABIS) were extracted with two methods for separate extraction of lipids and polar/semipolar metabolites and the extracts were then analyzed using two methods using an ultra-high-performance liquid chromatography quadrupole time-of-flight mass spectrometry (QTOFMS) and the data were processed using MZmine 2.53^39^as described below.

### Experimental model and subject details

ABIS is a general population prospective birth cohort designed to identify environmental and genetic factors associated with autoimmune diseases.^27^ A total of 1,435 ABIS infants had their HLA genotype sequenced. We selected children who later developed specific immune-mediated diseases, i.e., those subjects who later were diagnosed with either T1D, CD, IBD (Crohn's disease, Colitis ulcerosa), JIA or HT, and controls who remained healthy during the follow-up, matched for date of birth and sex (Table 1). The clinical parameters were similar across the different groups, with only birth weight showing significantly different values in those children who progressed to CD or HT later in life. The Swedish National Patient Register provided the diagnoses (https://www.socialstyrelsen.se/en/statistics-and-data/registers/national-patient-register/). CD diagnosis was determined only if the subjects had the diagnosis confirmed after their initial diagnosis. The gestational age, birth weight, or the type of delivery did not show statistically significant differences across the groups.

This study was performed in accordance with the Declaration of Helsinki. The ABIS study was approved by the Research Ethics Committees of the Faculty of Health Science at Linköping University, Sweden, 1997/96,287 and 2003/03–092 and the Medical Faculty of Lund University, Sweden.

#### Lipidomics and metabolomics

A total of 360 cord blood samples were randomized and analyzed as described below. . Quantification was performed using calibration curves and the identification was done with a custom database, with identification levels 1 and 2, based on Metabolomics Standards Initiative. Quality control was performed by analysing pooled quality control samples. In addition, a reference standard (NIST 1950 reference plasma), extracted blank samples and standards were analyzed as part of the quality control procedure.

#### Lipidomic analysis

A total of 360 cord blood samples were randomized and analyzed as described below. 10 μL of serum was mixed with 10 μL 0.9% NaCl and extracted with 120 μL of CHCl_3_: MeOH (2:1, v/v) solvent mixture containing internal standard mixture (c = 2.5 μg/mL; 1,2-diheptadecanoyl-*sn*-glycero-3-phosphoethanolamine (PE(17:0/17:0)), N-heptadecanoyl-D-erythro-sphingosylphosphorylcholine (SM(d18:1/17:0)), N-heptadecanoyl-D-erythro-sphingosine (Cer(d18:1/17:0)), 1,2-diheptadecanoyl-sn-glycero-3-phosphocholine (PC(17:0/17:0)), 1-heptadecanoyl-2-hydroxy-sn-glycero-3-phosphocholine (LPC(17:0)) and 1-palmitoyl-d31-2-oleoyl-sn-glycero-3-phosphocholine (PC(16:0/d31/18:1)) and, triheptadecanoylglycerol (TG(17:0/17:0/17:0)). The samples were vortexed and let stand on the ice for 30 min before centrifugation (9400 rcf, 3 min). 60 μL of the lower layer of was collected and diluted with 60 μL of CHCl_3_: MeOH. The samples were kept at −80°C until analysis.

The samples were analyzed using an ultra-high-performance liquid chromatography quadrupole time-of-flight mass spectrometry (UHPLC-QTOFMS from Agilent Technologies; Santa Clara, CA, USA). The analysis was carried out on an ACQUITY UPLC BEH C18 column (2.1 mm × 100 mm, particle size 1.7 μm) by Waters (Milford, USA). Quality control was performed throughout the dataset by including blanks, pure standard samples, extracted standard samples and control plasma samples. The eluent system consisted of (A) 10 mM NH_4_Ac in H_2_O and 0.1% formic acid and (B) 10 mM NH_4_Ac in ACN: IPA (1:1) and 0.1% formic acid. The gradient was as follows: 0–2 min, 35% solvent B; 2–7 min, 80% solvent B; 7–14 min 100% solvent B. The flow rate was 0.4 mL/min.

Data were processed using MZmine 2.^39^ Mass spectrometry data processing was performed using the open source software package MZmine 2.53.^40^ The following steps were applied in this processing: (i) Mass detection with a noise level of 100, (ii) Chromatogram builder with a minimum time span of 0.08 min, minimum height of 1000 and an *m*/*z* tolerance of 0.006 *m*/*z* or 10.0 ppm, (iii) Chromatogram deconvolution using the local minimum search algorithm with a 70% chromatographic threshold, 0.05 min minimum RT range, 5% minimum relative height, 1200 minimum absolute height, a minimum ration of peak top/edge of 1.2 and a peak duration range of 0.08–5.0, (iv), Isotopic peak grouper with an *m*/*z* tolerance of 5.0 ppm, RT tolerance of 0.05 min, maximum charge of 2 and with the most intense isotope set as the representative isotope, (v) Join aligner with an *m*/*z* tolerance of 0.009 or 10.0 ppm and a weight for of 2, an RT tolerance of 0.15 min and a weight of 1 and with no requirement of charge state or ID and no comparison of isotope pattern, (vi) Peak list row filter with a minimum of 10% of the samples (vii) Gap filling using the same RT and *m*/*z* range gap filler algorithm with an *m*/*z* tolerance of 0.009 *m*/*z* or 11.0 ppm, (vii) Identification of lipids using a custom database search with an *m*/*z* tolerance of 0.008 *m*/*z* or 8.0 ppm and an RT tolerance of 0.25 min. Identification of lipids was based on in house laboratory based on LC-MS/MS data on retention time and mass spectra. The identification was done with a custom database, with identification levels 1 and 2, i.e. based on authentic standard compounds (level 1) and based on MS/MS identification (level 2) based on Metabolomics Standards Initiative. Quality control was performed by analysing pooled quality control samples (with an aliquot pooled from each individual samples) together with the samples. In addition, a reference standard (NIST 1950 reference plasma), extracted blank samples and standards were analyzed as part of the quality control procedure.

#### Analysis of polar metabolites

40 μL of serum sample was mixed with 90 μL of cold MeOH/H2O (1:1, v/v) containing the internal standard mixture (Valine-d8, Glutamic acid-d5, Succinic acid-d4, Heptadecanoic acid, Lactic acid-d3, Citric acid-d4. 3-Hydroxybutyric acid-d4, Arginine-d7, Tryptophan-d5, Glutamine-d5, each at at c = 1 μgmL-1 and 1D4-CA,1D4-CDCA,1D4-CDCA,1D4-GCA,1D4-GCDCA,1D4-GLCA,1D4-GUDCA,1D4-LCA,1D4-TCA, 1D4-UDCA, each at 0.2 1 μgmL-1) for protein precipitation. The tube was vortexed and ultrasonicated for 3 min, followed by centrifugation (10000 rpm, 5 min). After centrifuging, 90 μL of the upper layer of the solution was transferred to the LC vial and evaporated under the nitrogen gas to the dryness. After drying, the sample was reconstituted into 60 μL of MeOH: H_2_O (70:30).

Analyses were performed on an Agilent 1290 Infinity LC system coupled with 6545 Q-TOF MS interfaced with a dual jet stream electrospray (dual ESI) ion source (Agilent Technologies, Santa Clara, CA, USA) was used for the analysis. Aliquots of 10 μL of samples were injected into the Acquity UPLC BEH C18 2.1 mm × 100 mm, 1.7-μm column (Waters Corporation)), fitted with a C18 precolumn (Waters Corporation, Wexford, Ireland. The mobile phases consisted of (A) 2 mM NH_4_Ac in H_2_O: MeOH (7:3) and (B) 2 mM NH_4_Ac in MeOH. The flow rate was set at 0.4 mLmin-1 with the elution gradient as follows: 0–1.5 min, mobile phase B was increased from 5% to 30%; 1.5–4.5 min, mobile phase B increased to 70%; 4.5–7.5 min, mobile phase B increased to 100% and held for 5.5 min. A post-time of 5 min was used to regain the initial conditions for the next analysis. The total run time per sample was 20 min. The dual ESI ionization source was settings were as follows: capillary voltage was 4.5 kV, nozzle voltage 1500 V, N2 pressure in the nebulized was 21 psi and the N2 flow rate and temperature as sheath gas was 11 Lmin-1 and 379°C, respectively. In order to obtain accurate mass spectra in MS scan, the *m*/*z* range was set to 100–1700 in negative ion mode. MassHunter B.06.01 software (Agilent Technologies, Santa Clara, CA, USA) was used for all data acquisition.

### Quantification and statistical analysis

#### Quantification

Quantification of lipids was performed using a 7-point internal calibration curve (0.1–5 μg/mL) using the following lipid-class specific authentic standards: using 1-hexadecyl-2-(9Z-octadecenoyl)-*sn*-glycero-3-phosphocholine (PC(16:0e/18:1(9Z))), 1-(1Z-octadecenyl)-2-(9Z-octadecenoyl)-sn-glycero-3-phosphocholine (PC(18:0p/18:1(9Z))), 1-stearoyl-2-hydroxy-sn-glycero-3-phosphocholine (LPC(18:0)), 1-oleoyl-2-hydroxy-sn-glycero-3-phosphocholine (LPC(18:1)), 1-palmitoyl-2-oleoyl-sn-glycero-3-phosphoethanolamine (PE(16:0/18:1)), 1-(1Z-octadecenyl)-2-docosahexaenoyl-sn-glycero-3-phosphocholine (PC(18:0p/22:6)) and 1-stearoyl-2-linoleoyl-sn-glycerol (DG(18:0/18:2)), 1-(9Z-octadecenoyl)-sn-glycero-3-phosphoethanolamine (LPE(18:1)), N-(9Z-octadecenoyl)-sphinganine (Cer(d18:0/18:1(9Z))), 1-hexadecyl-2-(9Z-octadecenoyl)-sn-glycero-3-phosphoethanolamine (PE(16:0/18:1)) from Avanti Polar Lipids, 1-Palmitoyl-2-Hydroxy-sn-Glycero-3-Phosphatidylcholine (LPC(16:0)), 1,2,3 trihexadecanoalglycerol (TG(16:0/16:0/16:0)), 1,2,3-trioctadecanoylglycerol (TG(18:0/18:0/18:)) and 3β-hydroxy-5-cholestene-3-stearate (ChoE(18:0)), 3β-Hydroxy-5-cholestene-3-linoleate (ChoE(18:2)) from Larodan, were prepared to the following concentration levels: 100, 500, 1000, 1500, 2000 and 2500 ng/mL (in CHCl3:MeOH, 2:1, v/v) including 1250 ng/mL of each internal standard.

Quantification of BAs was performed using a 7-point internal calibration curve using metabolites specified in key resources table. The identification was done with a custom data base, with identification levels 1 and 2, based on Metabolomics Standards Initiative. Quality control was performed by analysing pooled quality control samples (with an aliquot pooled from each individual samples) together with the samples. In addition, a reference standard (NIST 1950 reference plasma), extracted blank samples and standards were analysed as part of the quality control procedure.

#### Statistical analyses

Missing values were replaced by half of the minimum value. Metabolites with a relative standard deviation >30% in pooled QC samples were removed from further analysis for unsatisfactory analytical robustness. The metabolomics data was scaled and logarithmic transformed prior the statistical analysis to ensure normal distribution of the data.

#### Model-based metabolite clustering

Clustering of the ECs, lipidomic and metabolomics data obtained in this study was performed by using the ‘*mclust*’ R package (v.5.4.6). *Mclust* is a model-based clustering method, where the model performances are evaluated by the Bayesian Information Criterion (BIC). The models with the highest BICs were chosen.

#### Linear regression analysis

Linear regression analysis using Limma available from MetaboAnalyst 5.0 was used to estimate mean differences between the control and individual disease groups and to identify differentially expressed metabolites.^41^^,^^42^ A two-sided t-test was performed to calculate p values for each metabolite and multiple testing correction using the Benjamini-Hochberg method was applied to control the false discovery rate (FDR). The log-fold change in expression (logFC) between the groups was also calculated using Limma. Metabolites with p values less than 0.05 and adjusted P-values less than 0.05 were considered significant and further analyzed. Heatmaps were used to show the fold changes in metabolite levels between control and individual disease groups, where the control group was used as the baseline for the heatmap.

#### Pathway analysis

Pathway overrepresentation analysis was performed using the MetaboAnalyst 5.0 web platform using the Functional Analysis (MS Peaks)” module.^41^ For the input data for pathway analysis the complete high-resolution LC-MS spectral peak data obtained in negative ionization mode was used (mass tolerance of 10 ppm). A Welch’s t-test was performed to assess significant mean differences in the concentration of metabolites between cases and controls, and the whole input peak list with p values and T score was used for the pathway analysis. The relative significance of the overrepresented pathways against the background human scale metabolic model MNF (from MetaboAnalyst Mummichog package) and Kyoto Enzyclopedia of Genes and Genomes (KEGG) pathways [9] for *Homo sapiens* were estimated. The ‘Pathway Impact Scores’ were calculated by the metabolomics pathway analysis (MetPA) tool^43^ encoded in MetaboAnalyst 5.0.^41^^,^^44^

## Data Availability

• This paper does not report original code. • The metabolomics data reported in this paper will be shared by the lead contact upon request. • Any additional information required to reanalyze the data reported in this paper is available from the lead contact upon request and an appropriate institutional collaboration agreement. These data are not available to access in a repository owing to concern that the identity of patients might be revealed inadvertently.

## Supplemental information

Document S1. Tables S3 and S4

Table S1. Spearman correlations between metabolites and lifestyle parameters, related to Figure 1

Table S2. Linear regression model for lipids, adjusted for birth weight, gestational age, and maternal age, related to Figure 2
