# Supplementary material for: Cord serum metabolic signatures of future progression to immune-mediated diseases
Source: iScience. 2023 Feb 25;26(3):106268. doi: 10.1016/j.isci.2023.106268 (PMC10005901; doi:10.1016/j.isci.2023.106268)
Supplement: Document S1. Tables S3 and S4 [file mmc1.pdf]

iScience, Volume *26*

Supplemental information

Cord serum metabolic signatures of future progression to immune-mediated diseases

Tuulia Hyötyläinen, Bagavathy Shanmugam Karthikeyan, Tannaz Ghaffarzadegan, Eric W. Triplett, Matej Orešič, and Johnny Ludvigsson

Supplementary Material

Table S3. Linear regression model for polar metabolites, adjusted for birth weight, gestational age, and maternal age. Related to Figure 2.

	All autoimmune cases (not HT)			HT			JIA			IBD			T1D			Celiac disease		
**Metabolite**	**logFC**	**P.Value**	**adj.P.Val**	**logFC**	**P.Value**	**adj.P.Val**	**logFC**	**P.Value**	**adj.P.Val**	**logFC**	**P.Value**	**adj.P.Val**	**logFC**	**P.Value**	**adj.P.Val**	**logFC**	**P.Value**	**adj.P.Val**
7-Ketolithocholic acid*	**-0.48**	**0.0011**	0.11	-0.25	0.5490	0.85	-0.47	0.1650	0.91	**-1.03**	**0.0070**	0.18	-0.33	0.2623	0.86	**-0.40**	**0.0437**	0.48
Serine	**-0.44**	**0.0026**	0.13	-0.13	0.7490	0.88	-0.25	0.4546	0.91	0.09	0.8237	0.98	-0.55	0.0658	0.65	**-0.57**	**0.0043**	0.24
UDCA	**-0.41**	**0.0045**	0.13	-0.38	0.3578	0.77	-0.21	0.5261	0.91	-0.25	0.5113	0.98	-0.53	0.0747	0.65	**-0.44**	**0.0266**	0.42
Isovaleric acid.1	**0.41**	**0.0054**	0.13	0.28	0.4955	0.82	**0.82**	**0.0146**	0.53	**0.83**	**0.0309**	0.45	-0.04	0.8831	1.00	**0.41**	**0.0428**	0.48
Isovaleric acid	**0.35**	**0.0167**	0.29	-0.16	0.6967	0.88	0.53	0.1142	0.91	0.06	0.8826	0.98	0.16	0.5920	0.96	**0.47**	**0.0181**	0.42
tryptophan	**-0.35**	**0.0179**	0.29	-0.30	0.4639	0.82	-0.13	0.7083	0.99	-0.11	0.7672	0.98	-0.56	0.0601	0.65	-0.35	0.0850	0.53
THDCA	**0.31**	**0.0312**	0.44	-0.61	0.1400	0.63	0.32	0.3376	0.91	**1.10**	**0.0039**	0.18	0.36	0.2293	0.86	0.13	0.5057	0.74
dehydroepiandrosterone sulfate	-0.20	0.1694	0.50	**-1.55**	**0.0002**	0.02	-0.24	0.4857	0.91	-0.31	0.4108	0.98	-0.32	0.2764	0.86	-0.01	0.9532	0.95
1-Hexanol arabinosylglucoside.2	-0.08	0.5646	0.83	**-1.20**	**0.0037**	0.21	0.00	0.9971	1.00	0.17	0.6577	0.98	-0.52	0.0829	0.65	0.04	0.8499	0.90
LysoPE(18:0).1	0.18	0.2196	0.57	**1.11**	**0.0072**	0.26	0.32	0.3398	0.91	0.12	0.7540	0.98	0.38	0.1974	0.81	0.09	0.6569	0.82
LysoPE(18:0)	0.28	0.0530	0.45	**1.07**	**0.0097**	0.26	0.15	0.6551	0.99	0.04	0.9197	0.98	**0.63**	**0.0340**	0.65	0.29	0.1543	0.53
TaMCA	0.05	0.7087	0.84	**-1.03**	**0.0132**	0.26	0.62	0.0661	0.91	-0.26	0.5031	0.98	0.00	0.9892	1.00	-0.04	0.8503	0.90
Malic Acid	0.26	0.0728	0.45	**1.02**	**0.0140**	0.26	0.07	0.8278	0.99	**1.08**	**0.0047**	0.18	0.18	0.5362	0.96	0.18	0.3636	0.63
Isocaproic acid	0.28	0.0510	0.45	**0.90**	**0.0297**	0.48	0.32	0.3458	0.91	**1.09**	**0.0044**	0.18	0.22	0.4578	0.96	0.20	0.3093	0.61
Linolenic acid	-0.07	0.6243	0.83	**0.87**	**0.0363**	0.51	**-0.85**	**0.0116**	0.53	-0.02	0.9610	1.00	-0.29	0.3235	0.92	0.33	0.1020	0.53
LPC(16:0)	0.08	0.5876	0.83	**0.83**	**0.0449**	0.53	0.05	0.8900	0.99	0.11	0.7828	0.98	0.05	0.8680	1.00	0.07	0.7141	0.82
Fumaric acid	0.15	0.3024	0.57	**0.81**	**0.0497**	0.53	-0.03	0.9326	0.99	**1.02**	**0.0078**	0.18	0.15	0.6153	0.96	0.02	0.9131	0.95
TCDCA_S	0.07	0.6308	0.83	0.16	0.7019	0.88	**1.00**	**0.0032**	0.36	0.53	0.1672	0.98	0.02	0.9389	1.00	-0.26	0.1935	0.54
7-Ketolithocholic acid*.1	-0.23	0.1105	0.45	0.32	0.4444	0.82	**-0.79**	**0.0189**	0.53	0.11	0.7812	0.98	0.28	0.3459	0.96	-0.34	0.0862	0.53
Indole-3-propionic acid	0.17	0.2399	0.57	0.44	0.2904	0.72	**0.66**	**0.0514**	0.91	0.13	0.7337	0.98	0.13	0.6663	0.96	0.07	0.7334	0.82
Glyco-dihydroxy bile acid sulfate*.1	0.01	0.9266	0.97	0.26	0.5222	0.84	-0.08	0.8223	0.99	**-0.86**	**0.0248**	0.45	0.45	0.1332	0.67	0.13	0.5307	0.76
Allocholic acid*	-0.26	0.0760	0.45	0.16	0.6923	0.88	-0.06	0.8572	0.99	**-0.82**	**0.0319**	0.45	-0.18	0.5338	0.96	-0.17	0.3947	0.65
Put_3-Chlorothieno[2,3-b]thiophene-2-carbonyl chloride	-0.57	**0.0001**	**0.01**	-0.23	0.5819	0.87	-0.23	0.4940	0.91	**-0.80**	**0.0368**	0.46	**-0.86**	**0.0040**	0.44	**-0.48**	**0.0161**	0.42
put_Alcophosphamide fragments found)	-0.53	**0.0003**	**0.01**	0.16	0.6986	0.88	-0.42	0.2121	0.91	-0.18	0.6433	0.98	**-0.60**	**0.0445**	0.65	**-0.60**	**0.0026**	0.24

Pregnanetriol 3a-O-b-D-glucuronide	0.25	0.0842	0.45	0.70	0.0919	0.53	0.21	0.5299	0.91	-0.14	0.7128	0.98	0.14	0.6407	0.96	**0.44**	**0.0267**	0.42
Put_2-Hydroxyacetophenone sulfate	-0.16	0.2486	0.55284	-0.29	0.4850	0.82	-0.07	0.8462	0.99	0.16	0.6750	0.98	0.13	0.6503	0.96	**-0.40**	**0.0450**	0.48
C20:5	0.14	0.3423	0.63	-0.42	0.3161	0.74	-0.26	0.4396	0.91	0.15	0.6913	0.98	-0.13	0.6527	0.96	**0.40**	**0.0472**	0.48
Fructose	-0.22	0.1327	0.48	0.02	0.9556	0.98	-0.05	0.8765	0.99	0.21	0.5873	0.98	**-0.69**	**0.0203**	0.65	-0.16	0.4286	0.67
LPC(17:0)	0.21	0.1555	0.48	0.65	0.1165	0.59	-0.44	0.1875	0.91	-0.12	0.7528	0.98	**0.65**	**0.0287**	0.65	0.31	0.1171	0.53
CDCA	-0.24	0.1074	0.45	-0.29	0.4798	0.82	0.25	0.4521	0.91	-0.23	0.5551	0.98	**-0.59**	**0.0469**	0.65	-0.30	0.1399	0.53

Table S4. Linear regression model for HLA-conferred risk of T1D (in relation to reduced risk X-1) and celiac disease (in relation to low risk X0). (A) lipid and polar metabolite clusters, (B) selected metabolites associated with HLA-conferred risk of T1D. Related to Figure 6.

(A)

	**T1D risk**						**Celiac risk**			
		X0/X.-1	X1./X.-1	X2/.X.-1	P.Value			**X1/X0**	**X2/X0**	**P.Value**
Lipid clusters	LC1	-0.27	-0.33	-0.15	0.397		LC1	0.54	0.03	0.392
	**LC2**	-0.76	-0.36	-1.03	**0.018**		LC2	-0.08	-0.40	0.425
	LC3	-0.68	-0.42	-0.48	0.130		LC3	0.08	0.14	0.922
	LC4	-0.71	-0.42	-0.53	0.066		LC4	0.24	0.17	0.848
	LC5	-0.14	-0.25	-0.33	0.901		LC5	0.12	-0.33	0.376
	LC6	-0.45	-0.29	-0.93	0.362		LC6	-0.03	-0.21	0.775
	LC7	0.16	0.04	-0.01	0.961		LC7	0.65	0.40	0.338
	LC8	-0.05	0.00	0.31	0.779		LC8	0.48	0.42	0.427
Polar metabolite clusters	PC1	0.13	0.13	0.20	0.404		PC1	0.37	0.25	0.689
	**PC2**	-0.43	-0.26	-0.13	**0.041**		PC2	-0.46	-0.33	0.545
	PC3	-0.11	-0.16	-0.02	0.787		PC3	0.15	-0.15	0.705
	**PC4**	-0.92	-0.62	-0.44	**0.015**		PC4	-0.74	-0.39	0.270
	PC5	-0.42	-0.30	0.01	0.340		PC5	-0.33	0.19	0.381
	PC6	-0.08	0.28	0.65	0.219		PC6	0.02	0.33	0.515
	PC7	0.60	0.06	0.14	0.246		PC7	0.46	0.16	0.603
	PC8	0.15	0.33	0.46	0.702		PC8	0.85	0.65	0.109
	PC9	0.34	0.00	0.37	0.694		PC9	0.14	-0.03	0.906
	PC10	-0.33	-0.56	-0.28	0.420		PC10	-0.68	-0.61	0.173
	PC11	-0.69	-0.76	-0.40	0.052		PC11	-0.59	-0.28	0.441
	PC12	0.14	-0.10	0.33	0.818		PC12	0.34	0.34	0.594

(B)

	T1D risk	X0/X.-1	X1/X.-1	X2/X.-1	P.Value	adj.P.Val
Polar metabolites	C22:6	-1.30	-1.11	-1.53	**0.0043**	0.471
	tryptophan	-1.21	-1.10	-1.43	**0.0085**	0.471
	TbMCAisomer	-0.69	-0.01	0.62	**0.0151**	0.558
	TCDCA	0.95	0.29	1.03	**0.0368**	0.639
	Arachidonic acid	-0.94	-1.01	-1.22	**0.0386**	0.639
	C18:2	-1.10	-0.95	-0.74	**0.0483**	0.639
	3-Indoleacetic acid	0.44	0.46	-0.62	**0.0497**	0.639
Lipids	PC(38:5)	-1.15	-0.59	-1.48	**0.0059**	0.183
	PC(38:5).1	-1.34	-0.80	-1.13	**0.0065**	0.183
	PI(38:7)	-1.06	-0.70	-1.61	**0.0075**	0.183
	LPC(20:4)	-1.04	-0.31	-1.17	**0.0119**	0.183
	PC(38:3)	-1.28	-0.80	-0.74	**0.0123**	0.183
	SM(d36:2)	-1.27	-0.93	-1.00	**0.0127**	0.183
	PC(35:1)	-1.18	-0.40	-0.67	**0.0132**	0.183
	PC(36:2).1	-1.26	-0.78	-0.67	**0.0135**	0.183
	PC(38:4).1	-0.87	0.11	-0.52	**0.0136**	0.183
	SM(d38:2)	-1.25	-0.67	-0.66	**0.0139**	0.183
	PC(O-40:5)	-1.23	-0.80	-1.12	**0.0152**	0.183
	PC(36:1)	-1.18	-0.53	-0.41	**0.0155**	0.183
	CE(20:4)	-1.18	-0.61	-1.08	**0.0168**	0.183
	SM(d34:2)	-1.23	-0.84	-0.99	**0.0179**	0.183
	PC(38:4).2	-1.09	-0.81	-1.39	**0.0180**	0.183
	PC(40:4)	-0.78	0.20	-0.32	**0.0184**	0.183
	PC(40:7)	-0.96	-0.39	-1.29	**0.0194**	0.183
	PC(O-38:4)	-0.99	-0.21	-0.91	**0.0203**	0.183
	SM(d18:1/24:4)	-1.19	-0.64	-0.60	**0.0206**	0.183
	PI(44:4)	-1.21	-0.77	-0.96	**0.0211**	0.183
	PC(O-38:5)	-0.88	0.01	-0.62	**0.0214**	0.183

	SM(d18:2/14:0)	-1.19	-0.95	-0.83	**0.0214**	0.183
	DG(34:2)	-1.08	-0.69	-0.21	**0.0246**	0.196
	PC(42:8)	-1.17	-0.65	-0.70	**0.0258**	0.196
	PC(38:6)	-1.12	-0.58	-1.04	**0.0260**	0.196
	PC(37:3)	-1.15	-0.56	-0.72	**0.0274**	0.198
	PC(37:4)	-0.93	-0.47	-1.29	**0.0292**	0.203
	LPC(20:5)	-0.99	-0.32	-0.98	**0.0305**	0.205
	PC(36:4)	-0.88	-0.48	0.17	**0.0360**	0.213
	PC(O-36:3)	-1.07	-0.44	-0.49	**0.0369**	0.213
	PC(32:2)	-1.07	-0.56	-1.01	**0.0385**	0.213
	PC(16:0e/18:1(9Z))	-1.12	-0.64	-0.69	**0.0393**	0.213
	LPE(20:4)	-0.80	-0.35	-1.26	**0.0403**	0.213
	PC(O-38:6)	-1.02	-0.45	-0.98	**0.0415**	0.213
	PC(38:4)	-0.87	-0.07	-0.53	**0.0438**	0.213
	LPC(22:6)	-0.73	0.01	-0.83	**0.0445**	0.213
	LPC(18:2)	-0.95	-0.28	-0.85	**0.0448**	0.213
	SM(40:2)	-1.07	-0.52	-0.54	**0.0449**	0.213
	PC(36:4).1	-0.88	-0.69	-1.35	**0.0450**	0.213
	SM(d42:3).1	-1.10	-0.66	-0.67	**0.0454**	0.213
	PC(40:8)	-0.53	-0.08	-1.17	**0.0490**	0.217
